# Glycation of Nail Proteins: From Basic Biochemical Findings to a Representative Marker for Diabetic Glycation-Associated Target Organ Damage

**DOI:** 10.1371/journal.pone.0120112

**Published:** 2015-03-17

**Authors:** Antoine Sadiki Kishabongo, Philippe Katchunga, Elisabeth H. Van Aken, Reinhart Speeckaert, Sabrina Lagniau, Renaat Coopman, Marijn M. Speeckaert, Joris R. Delanghe

**Affiliations:** 1 Department of Laboratory Medicine, Catholic University of Bukavu, Bukavu, Democratic Republic of the Congo; 2 Department of Internal Medicine, Catholic University of Bukavu, Bukavu, Democratic Republic of the Congo; 3 Department of Ophthalmology, Sint-Elisabeth Ziekenhuis, Zottegem, Belgium; 4 Department of Dermatology, Ghent University Hospital, Ghent, Belgium; 5 Department of Clinical Chemistry, Ghent University Hospital, Ghent, Belgium; 6 Department of Dentistry, Ghent University Hospital, Ghent, Belgium; 7 Department of Nephrology, Ghent University Hospital, Ghent, Belgium; Swiss Institute of Bioinformatics, SWITZERLAND

## Abstract

**Background:**

Although assessment of glycated nail proteins may be a useful marker for monitoring of diabetes, their nature and formation are still poorly understood. Besides a detailed anatomical analysis of keratin glycation, the usefulness of glycated nail protein assessment for monitoring diabetic complications was investigated.

**Methods:**

216 patients (94 males, 122 females; mean age ± standard deviation: 75.0 ± 8.7 years) were enrolled. Glycation of nail and eye lens proteins was assessed using a photometric nitroblue tetrazolium-based assay. Following chromatographic separation of extracted nail proteins, binding and nonbinding fractions were analyzed using one-dimensional gel electrophoresis. Using a hand piece containing a latch-type-bur, a meticulous cutting of the nail plate into superficial and deep layers was performed, followed by a differential analysis of fructosamine.

**Results:**

Using SDS PAGE, four and two bands were identified among the nonglycated and glycated nail fraction respectively. Significantly lower fructosamine concentrations were found in the superficial nail layer (mean: 2.16 ± 1.37 μmol/g nails) in comparison with the deep layer (mean: 4.36 ± 2.55 μmol/g nails) (P<0.05). A significant higher amount of glycated eye lens proteins was found in diabetes mellitus patients (mean: 3.80 ± 1.57 μmol/g eye lens) in comparison with nondiabetics (mean: 3.35 ± 1.34 μmol/g eye lens) (P<0.05). A marked correlation was found between glycated nail and glycated eye lens proteins [y (glycated nail proteins) = 0.39 + 0.99 x (eye lens glycated proteins); r^2^ = 0.58, P<0.001]. The concentration of glycated eye lens proteins and the HbA1c level were found to be predictors of the concentration of glycated nail proteins.

**Conclusions:**

Glycation of nail proteins takes place in the deep layer of finger nails, which is in close contact with blood vessels and interstitial fluid. Glycation of nail proteins can be regarded as a representative marker for diabetic glycation-associated target organ damage.

## Introduction

In sub-Saharan Africa, the assessment of glycated proteins in human finger nails may be a useful marker for monitoring of diabetes in comparison with the classic glycemia markers [measurement of plasma glucose concentration, oral glucose tolerance test (OGTT) and determination of HbA1c] due to a less critical preanalytical phase and low reagent costs [1]. However to the best of our knowledge, the nature and formation of glycated nail proteins are still poorly understood. As human nails do not contain blood vessels and are metabolically inactive, the high degree of protein glycation in healthy and diabetic subjects is not easy to understand. Therefore, a detailed anatomical analysis of keratin glycation is necessary. In addition, it should be interesting to study if the degree of glycation in finger nails is representative for the glycation of clinically more important target organs, such as the eye lens. This biochemical exploration could further confirm the usefulness of glycated nail protein assessment for monitoring diabetic complications.

In diabetes mellitus, hyperglycemia causes a non-enzymatic glycation of free amino groups of proteins. This is called the Maillard reaction, which can be subdivided into three steps. The early stage consists of the formation of Schiff’s bases, which are enzymatic intermediates where the amino group, such as the terminal group of a lysine molecule reversibly reacts with the aldehyde group of glucose (double bond between the carbon atom of the glucose and the nitrogen atom of the lysine molecule). The next step comprises a re-arrangement from the Schiff’s base, leading to the formation of Amadori products (the hydrogen atom from the hydroxyl group adjacent to the carbon-nitrogen double bond moves to bond to the nitrogen, leaving a ketone). In the last phase, oxidiation of the Amadori products leads to the formation of irreversible advanced glycation end-products (AGEs), which are widely recognized as the major cause of secondary diabetic complications [2–5]. The reactivity of the ε-amino group of a lysine residue or the α-amino group of an N-terminal residue is the preferential target for glycation in many human tissues [6–8]. Human serum albumin, IgG, histone proteins and crystallins are examples of lysine-rich proteins. Although AGEs affect nearly every type of cell and molecule in the body, accumulation of AGEs in tissues with a high amount of lysine-rich residues is correlated with increased tissue stiffness in arteries, lens, skin and tendon. Protein glycation affects cellular and extracellular proteins (e.g. collagen, crystallin, albumin, hemoglobin) [6,9–11]. Besides the lysine residue, glycation also takes place at the side chains of arginine, histidine, tryptophan and cysteine residues [3].

Recently, the assessment of nail protein glycation has been presented as a simple alternative for diagnosing diabetes mellitus in remote areas [1]. Higher concentrations of glycated nail proteins are observed in patients with diabetic complications, such as diabetic nephropathy and diabetic retinopathy. Looking at the anatomy of the nail, different compartments can be distinguished: the nail plate, the nail matrix, the nail bed and the surrounding soft tissues with the innervation and vasculature of the distal phalanx. The nail plate is a modified form of the stratum corneum lying on two structures: the nail matrix (15–25%) and the nail bed (75–85%). The deeper layers of the nail plate are formed by the intermediate region of the nail matrix and by the nail bed. The dorsal section of the nail matrix is responsible for the formation of the superficial layers of the nail plate. The growth of the nail plate is characterized by a longitudinal elongation, using the lunula or proximal nail fold as a reference structure. The blood supply of the fingers if fulfilled by radial and ulnar arteries, forming deep and superficial palmar arcades with branches aligned with the phalanges [12,13]. Both nail and eye lens tissues are composed of intermediate filament proteins, also called keratins and crystallins respectively [14–20]. The human nail plate is mainly made up of 10–20% *soft* or *cyto*keratins (epithelial type) and 80–90% *hard* or *trichocytic* keratins (hair type) [21–24]. The difference between both kinds of keratins is the sulfur content in their non-α-helical head and tail domains, which is considerably higher in hair keratins and responsible for the extraordinary high degree of filamentous cross-linking by keratin-associated proteins [25]. The keratins are characterized by a broad molecular diversity and have a molecular weight of 44–66 kDa [14,26]. The process of nail protein glycation can take six to nine months, as this is the time interval for a complete human nail replacement [24]. This means that the glycation level in the human nail is submitted to fluctuations as the nail is progressively replaced during this period. On the other hand, the transparent eye lens is composed of crystallins, belonging to two protein superfamilies: the α- and βγ-crystallins [27]. As those crystallins are subject to life-long glycation, accumulation of AGEs in the eye lens will play an important role in diabetic cataract by formation of high-molecular-weight aggregates and by eye lens protein aggregation [9,28–30]. This also means that in contrast to the human nail, the accumulation of AGEs in the eye lens occurs more continuously over time without influence of tissue replacement, as the content of the lens is not replaced over a life time.

## Materials and Methods

### Ethics Statement

The study was approved by the local ethics committee (Ghent University hospital BUN: B670201215602). The study complied with the World Medical Association Declaration of Helsinki regarding ethical conduct of research involving human subjects. All subjects provided written informed consent prior to participation.

### Patients

Two hundred sixteen patients (94 males, 122 females; mean age ± standard deviation: 75.0 ± 8.7 years), who underwent cataract surgery in Belgium, were enrolled in this study. Among this group, 29.2% patients had diabetes mellitus type 2 and 70.8% patients were non-diabetics. The treatment of the diabetic patient group consisted of a mixture of diabetes drugs: metformin, sulfonylureas, glucagon-like peptide-1 receptor agonists, dipeptidyl peptidase 4 inhibitors and insulin. Following eye lens extraction, eye lens fragments were collected, centrifuged (3,000 g, 10 min, room temperature) and stored at -20°C. In a subgroup of 51 patients (18 males, 33 females; mean age ± standard deviation: 75.7 ± 8.2 years; 35 diabetes mellitus type 2 patients and 16 non-diabetics), finger nail clippings were collected prior to surgery.

### Fructosamine and HbA1c analysis

Fructosamine (1-amino-1-deoxy-D-fructose) is formed by a non-enzymatic reaction of the carbonyl group of glucose with an amino group of circulating serum proteins. The concentration of fructosamine is influenced by the concentration and profile of the different serum proteins, with glycated albumin as the most important component (accounts for ± 90% of the glycated serum proteins) [31]. Fructosamine was analyzed on both eye lens and nail fragments. After weighing, the nail clippings were transferred into a standard 10 mm pathway cuvette. One mL fructosamine reagent [0.25 mmol/L nitro blue tetrazolium (NBT) (Sigma, St. Louis, MO, USA) in a 0.1 mol/L sodium carbonate/bicarbonate buffer (pH: 10.3), containing 0.1% Triton X-100 (Fluka, St. Louis, MO, USA)], was added to the clippings. Following incubation (37°C, 60 min), photometric readings occurred at 530 nm in a UV-1800 photometer (Shimadzu, Kyoto, Japan) [32]. A commercial fructosamine standard (Roche, Mannheim, Germany) was used for standardizing the assay. Results were expressed as μmol of fructosamine per g of nail. Similarly, the glycation analysis of eye lens fragments (± 10 mg) was carried out, using an identical procedure after thawing at room temperature, weighing and incubation (37°C, 20 min) and then using the fructosamine reagent [0.25 mmol/L NBT (Sigma, St. Louis, MO, USA) in a 0.1 mol/L sodium carbonate/bicarbonate buffer (pH: 10.3)]. Results were expressed as μmol of fructosamine per g of eye lens.

The variability in size of nail clipping fragments is not a critical factor in the analysis. As already demonstrated in our previous paper [1], the mean within-run coefficient of variation (CV) for different finger nail fragments (0.3–2.8 mg per fragment) was 8.97%, which was comparable with the magnitude of the method’s CV (11%). In a similar way the effect of the ‘nail dose’, expressed as the amount of glycated nail protein/mg nail, was negligible with a within-run CV of 13%.

The fructosamine assay used in this study is different from the serum or plasma fructosamine assays. In plasma, reducing substances such as uric acid and ascorbic acid may interfere with the test result. However, one cannot compare the plasma/serum matrix and the nail matrix. In plasma, the protein concentration is “only” ± 7% (70 g/L) (in nail: ± 85%) and the exposure time of the plasma proteins to glucose is 2 to 3 weeks (in nails: 6–9 months). In plasma, the relative volume of the water compartment is ± 90% (containing 40–70 mg/L uric acid and ± 15 mg/L ascorbic acid), whereas in nail clippings, the water concentration is very small (only a few %). So, the concentration ratio of interfering substances:protein is negligible in a nail matrix (as compared to human serum or plasma). In case of human nail clippings, we are almost dealing with an ideal matrix for fructosamine testing: a pure protein matrix in absence of interfering substances.

Haemoglobin A1c (HbA1c) was assayed on ethylenediaminetetraacetic acid (EDTA) blood specimens using a Menarini 8160 high-performance liquid chromatography (HPLC) system (Menarini, Firenze, Italy).

### Nail proteins extraction

The extraction of nail keratins was carried out by the Shindai method [33]. Nail clippings from diabetic and nondiabetic individuals were collected and washed with ethanol before delipidization into the mixture chloroform/methanol (2:1, V/V, Merck, Darmstadt, Germany) for 24h. The extraction of nails was done using a solution containing 25 mmol/L Tris-HCl, pH: 8.5, 2.6 mol/L thiourea, 5 mol/L urea (Bio-Rad Laboratories Inc., Richmond, CA, USA) and 5% 2-mercaptoethanol (2-ME, Fluka, St. Louis, MO, USA) at 50°C for 3 days. The mixture was filtered and centrifuged at 15,000 *g* (20 min, room temperature). The obtained supernatant was used as nail protein extract.

### Analysis of superficial and deep layers

In order to study analytical interferences of the fructosamine reaction in nail clippings in detail, eight samples of nail clippings (4 diabetes mellitus type 2 patients and 4 nondiabetics) with known fructosamine concentrations, were sent to the Department of Dentistry of the Ghent University Hospital for a meticulous cutting into a powder. A hand piece (Rodeq WA-56 A) containing a latch-type-bur (Mani stainless steel bur, length 22 mm, size #2) was used to obtain very small pieces. During the preparation no water-cooling was used. The rotation speed was kept at a minimum to avoid thermal reactions. Following grinding, a powder was obtained. An amount of 25 mg nail powder was weighed. Consecutively, the nail powder was extracted using 1 mL phosphate buffered saline (0.1 mol/L) for 24 h at room temperature (20°C). The extract was then analyzed for uric acid using a commercial colorimetric method (Roche Diagnostics, Mannheim, Germany), which utilizes the enzyme uricase to oxidize uric acid. This method eliminates the interference intrinsic to chemical oxidation. The sample was initially incubated with a reagent mixture containing ascorbate oxidase and a clearing system. Upon addition of the starter reagent, oxidation of uric acid by uricase began. The peroxide reacted in the presence of peroxidase (POD), TOOS [N-ethyl-N-(2-hydroxy-3-sulfopropyl)-3-methyl aniline] and 4-aminoantipyrine to form a quinone-imine dye. The color intensity of the quinone-diimine formed was directly proportional to the uric acid concentration and was determined by measuring the increase in absorbance at 552 nm on a Cobas 8000 analyser. In all samples investigated, the uric acid concentration was undetectable (< 0.05 mg/dL extract), indicating that the uric acid concentration in the nail clippings was less than 40 μg/250 mg nails (210 mg protein). In other words, the interference caused by uric acid was negligible.

### Boronate affinity chromatography

The extracted nail proteins were transferred into a 2 mL boronate affinity column [Glass Econo-Column (Bio-Rad Laboratories Inc., Richmond, CA, USA)] to undergo a chromatographic separation into glycated nail proteins and nonglycated nail proteins. The wash buffer consisted of 0.25 mol/L ammonium acetate, containing 0.05 mol/L magnesium chloride, pH: 8.3. The elution buffer consisted of 0.1 mol/L Tris-HCl, containing 0.2 mol/L sorbitol (D-Glucitol, Sigma Aldrich, St. Louis, MO, USA) and 0.05 mol/L EDTA [34]. The concentration of proteins was assayed by the pyrogallol red-molybdate method on a Cobas 8000 analyzer [35]. The percentage of the glycated protein fraction was calculated from the protein content of both fractions. Following chromatography, binding and nonbinding fractions were analyzed using electrophoresis.

### Gel electrophoresis

The extracted reduced proteins collected from boronate affinity chromatography were analyzed using one-dimensional sodium dodecyl sulfate-polyacrylamide gel electrophoresis (SDS-PAGE, Sigma-Aldrich, St. Louis, MO, USA). The protein content in keratinized structures (including nails) is approximately 80% of the total mass. Following extraction, the protein concentration was assayed according to Orsonneau [35]. For nail extracts, we applied 5 μL of solution containing 20 g protein/L. So ± 100 μg of protein was applied. For the boronate eluates, we could reach a similar loading for the non-glycated part. Due to the elution step and the relative small amount of glycated proteins present in the specimen, a similar volume was applied containing ± 1 g protein/L. A 12% resolving acrylamide gel was used. For stacking, a 4% polyacrylamide gel was used. The electrophoresis buffer consisted of 0.025 mol/L Tris, 0.092 mol/L glycine, and 0.1% SDS. Following electrophoresis (200V, 1h), the proteins in the gel were visualized by Coomassie brilliant blue R-250 (Bio-Rad Laboratories Inc., Richmond, CA, USA) and destained in 10% acetic acid and 40% ethanol for 1h. The molecular mass of the different fractions, expressed in kDa, was determined using standard gel reference.

### Analysis of superficial and deep layers

Twelve samples of nail clippings (4 diabetes mellitus type 2 patients and 8 nondiabetics), with known fructosamine concentrations, were sent to the Department of Dentistry of the Ghent University Hospital for a meticulous cutting into superficial and deep layers, according to the anatomical structure of nail tissue. A hand piece (Rodeq WA-56 A) containing a latch-type-bur (Mani stainless steel bur, length 22 mm, size #2) removed the upper surface of the right side of the finger nail. Half of the upper surface of the right side of the finger nail was removed using pencil-like movements of the bur. As the nail plate thickness ranged from 0.25 to 0.50 mm, the deeper layer of the nail plate was reached at 0.12–0.25 mm. Using an analytical balance, the meticulous separation between the superficial and deeper layer was accomplished. When the procedure was finished, the prepared right side of the finger nail was fixed manually. The same procedure was used for the untreated left side. During the preparation no water-cooling was used. The rotation speed was kept at a minimum to avoid thermal reactions. Afterwards the roughness of the prepared upper surface was polished using a periodontal scaler type S204 (Department of Dentistry, Ghent University Hospital, Belgium). Following weighing, a differential analysis of fructosamine was performed in both superficial and deep fractions.

### Statistics

Statistical analyses were performed using MedCalc (MedCalc, Mariakerke, Belgium). Values are expressed as mean ± standard deviation (SD). The normality of the data distribution was evaluated by Kolmogorov-Smirnov test. We used the F-test for assessing equality of variances of the data distributions. Differences between groups were evaluated using the Student’s t-test. Pearson correlation coefficients were used to explore the relationships. Multiple linear regression analysis was performed with glycated nail protein as dependent variable. A P value <0.05 was considered a priori to be statistically significant.

## Results

**Fig 1:**
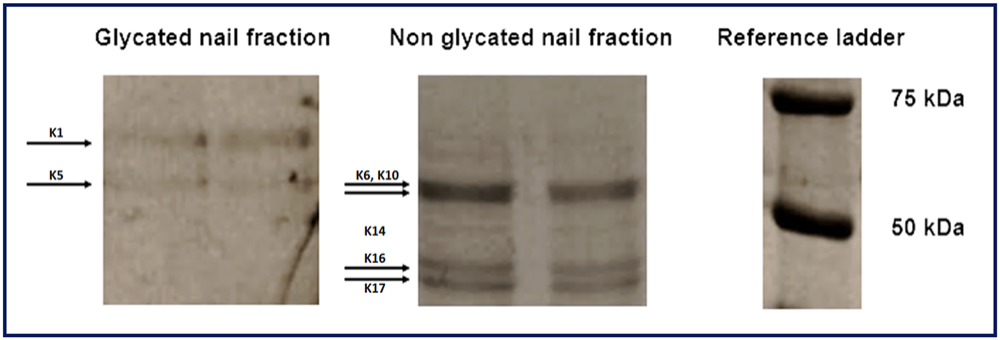
Sodium dodecyl sulfate-polyacrylamide gel electrophoresis (SDS-PAGE) of nail proteins after boronate affinity chromatography (extracted keratins with different molecular mass, ranging from 46 to 67 kDa).

Following nail protein extraction and boronate affinity chromatography, protein patterns of glycated and nonglycated nail fractions were compared. Using one-dimensional SDS PAGE, four major bands were identified among the nonglycated nail fraction. In the glycated nail fraction, only two bands were observed. A typical electrophoretogram is illustrated in Fig. 1. Similar to other glycated protein fractions (e.g. HbA1c), the glycated nail protein fraction represents only a minor part of the total nail protein extract eluted from boronate affinity chromatography: e.g. 5.1% of the total amount of nail proteins was found to be glycated in a patient with diabetes mellitus type 2.

**Fig 2:**
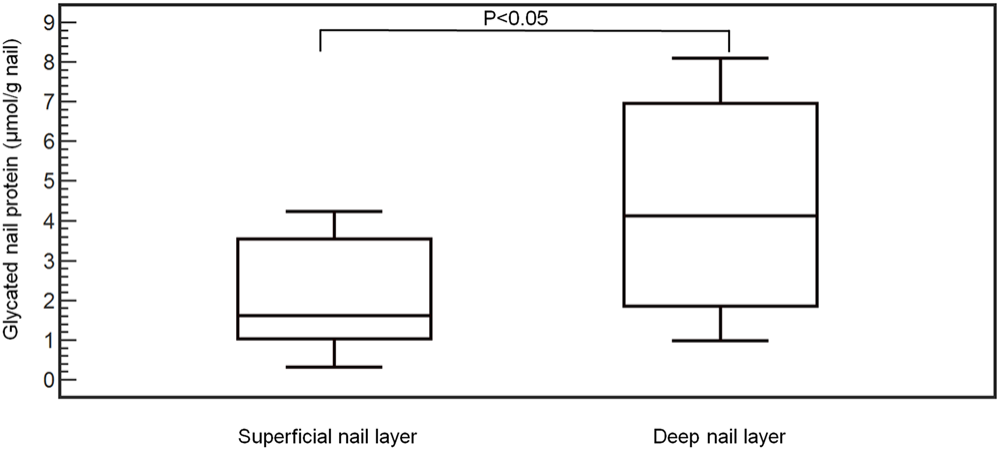
Glycated nail protein concentration between deep and superficial layers of the human finger nail (n = 12).

**Fig 3:**
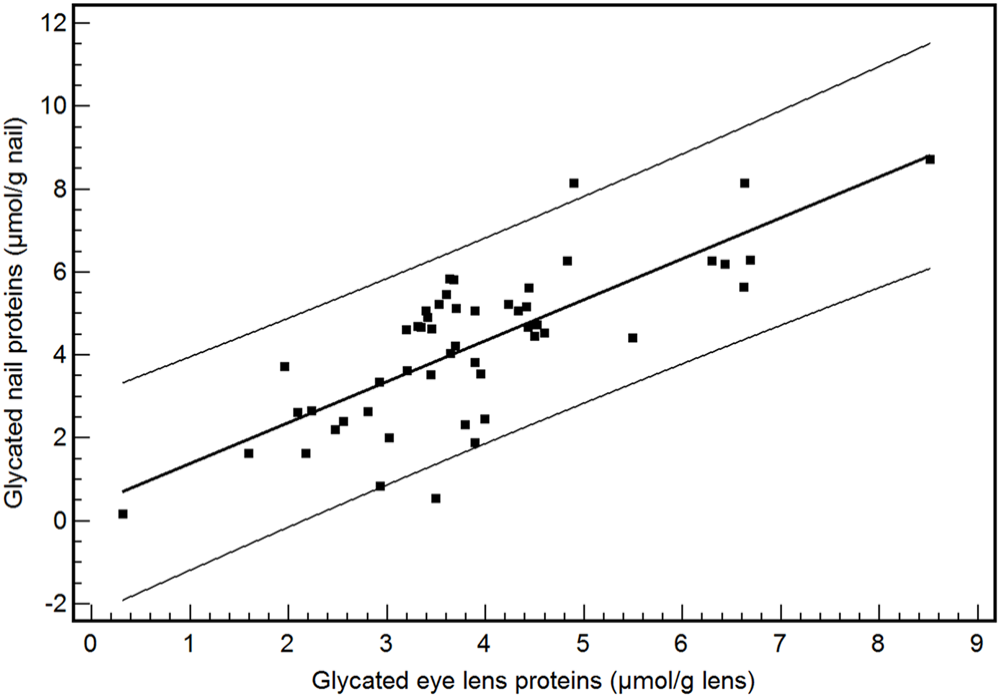
Correlation between glycated nail proteins and glycated eye lens proteins in the combined group of diabetes patients and nondiabetics (n = 51). The equation of linear regression is y (glycated nail proteins) = 0.39 + 0.99 x (glycated eye lens proteins) (r2 = 0.58; P<0.001).

**Fig 4:**
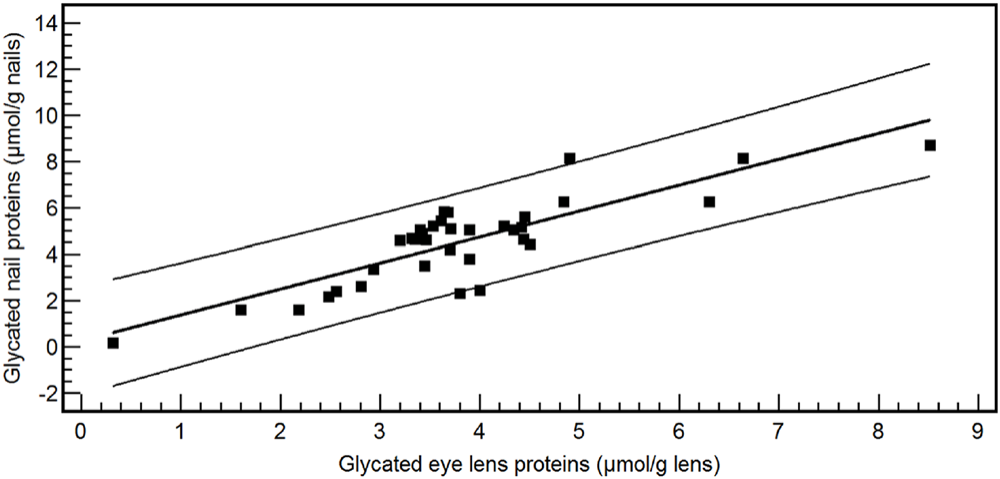
Correlation between glycated nail proteins and glycated eye lens proteins in the group of diabetes patients (n = 35). The equation of linear regression is y (glycated nail proteins) = 0.26 + 1.12 x (glycated eye lens proteins) (r2 = 0.71; P<0.001).

As already demonstrated in our previous work, the concentration of glycated nail proteins is significantly higher in patients with diabetes mellitus type than in nondiabetics [1]. However in this study, we showed that after meticulous cutting of nail clippings into superficial and deep layers (n = 12), a significantly lower concentration of fructosamine was found in the superficial layer of the nail (mean: 2.16 ± 1.37 μmol/g nails) in comparison with the deep layer of the nail (mean: 4.36 ± 2.55 μmol/g nails) (P<0.05) (Fig. 2). Moreover, the measurement of glycated eye lens proteins revealed a significant difference between diabetes mellitus type 2 patients (n = 63, mean: 3.80 ± 1.57 μmol/g eye lens) and nondiabetics (n = 153, mean: 3.35 ± 1.34 μmol/g eye lens) (P<0.05). In a subgroup of 51 cataract patients, the concentration of glycated nail and eye lens proteins was determined. A marked correlation was found between glycated nail proteins and glycated eye lens proteins [y (glycated nail proteins) = 0.39 + 0.99 x (glycated eye lens proteins); r^2^ = 0.58, P<0.001] (Fig. 3). This correlation was even stronger in diabetes mellitus type 2 patients [n = 35, y (glycated nail proteins) = 0.26 + 1.12 x (glycated eye lens proteins), r^2^ = 0.71, P<0.001] than in nondiabetics [n = 16, y (glycated nail proteins) = 0.19 + 0.83 x (glycated eye lens proteins), r^2^ = 0.56, P = 0.001] (Fig. 4). By using multiple regression analysis (Table 1), the concentration of glycated nail proteins was found to be a predictor of the concentration of glycated lens proteins and the HbA1c level. Age and sex did not reach significance in this model.

**Table 1:** Multiple regression model with glycated nail proteins as dependent variable.

Parameter	Independent variable	β (SE)	P
Glycated nail proteins	Age (years)	-0.0227 (-1.443)	NS
(μmol/g eye lens) r2 =	Sex	-0.3773 (-1.379)	NS
0.66	HbA1c (mmol/mol)	-0.0423 (-2.545)	= 0.01
	Glycated eye lens proteins (μmol/g nail)	0.6591 (0.0733)	<0.0001

## Discussion

As the protein content in keratinized structures (including nails) is approximately 80% of the total mass, slow glycation of nail proteins is observed in healthy persons [21,36,37]. However in case of diabetes mellitus, an increased concentration of glycated nail proteins is detected, explaining its usefulness as a diagnostic marker of this metabolic disorder of the carbohydrate metabolism [1,38]. Using SDS-PAGE, we identified in this study four bands in the nonglycated nail protein fraction and two bands in the glycated protein fraction, representing different epithelial keratins of the nail plate, with molecular masses ranging from 46 to 67 kDa [22,23,39–41]. The epithelial keratins K1, K5, K6, K10, K14, K16 and K17 are expressed in the nail plate, whereas the nail bed expresses the suprabasal keratins (K6, K16 and K17) and the nail matrix the keratins K1, K5, K10 and K14 [26,42]. As the reported keratins contain a significant proportion of lysine residues [43], those nail proteins are potential targets of glycation. The finding of increased fructosamine levels in finger nail clippings from diabetes mellitus patients confirms that glycated nail proteins could function as a potential diagnostic/prognostic marker of diabetes mellitus [44].

**Fig 5:**
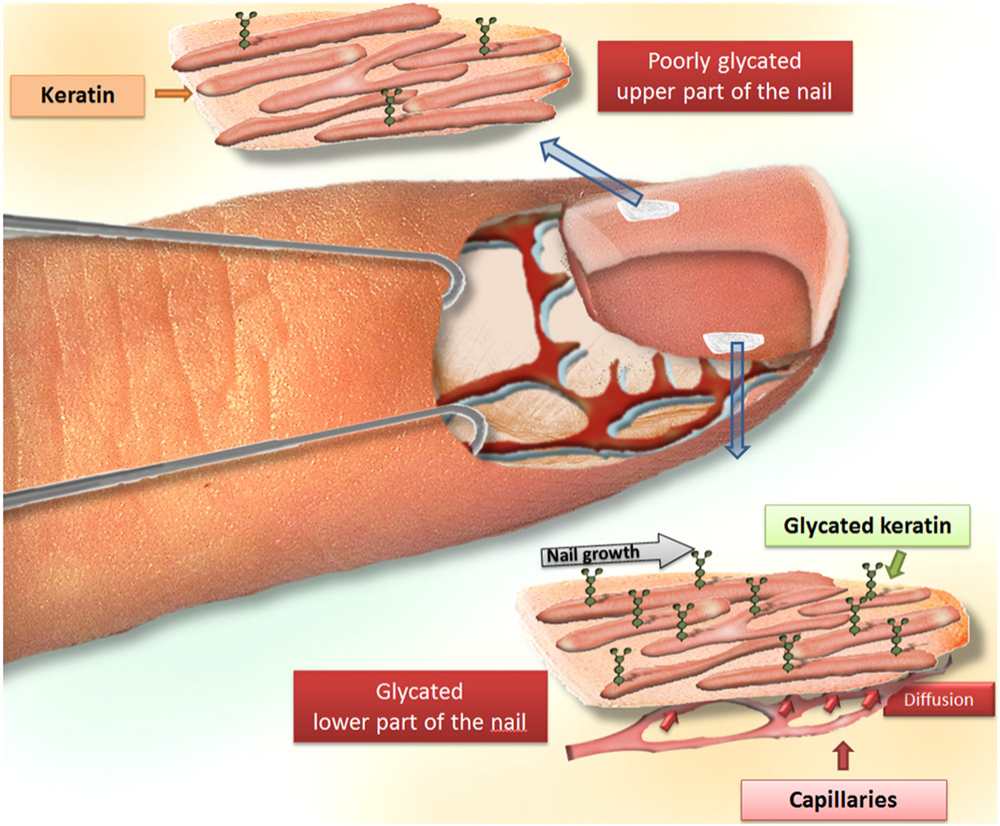
Illustration of the diffusion process of glucose from the deep part to the superficial part of the nail, explaining the differential concentration of glycated proteins in both layers of the human finger nail.

In spite of the great interest of protein glycation in the diagnosis of diabetes mellitus, little attention has been paid to the glycation process occurring in nail tissues. The analysis of glycated proteins from deep and superficial layers of the nail contributes to the understanding of glycated keratin formation. The significant higher fructosamine concentration in the deep layer than in the superficial layer of the human finger nail suggests that keratin glycation occurs in the deep layer of nails. The human nail structure is an appendage of the integument and forms a continuous and integrated structure with the skin, consisting of a nail bed, a nail matrix and a nail plate. The nail bed is a deep membrane with nerves, lymph vessels and blood vessels [26]. As nail proteins are exposed to glucose, originating from the blood and the extracellular fluid through its nail bed part, glycation of nail keratins can take place. Because of the complex morphological structure of nails (abundance of disulfide crosslinks), the diffusion of glucose from the deep part to the superficial part occurs very slow, explaining the differential concentration of glycated proteins in both layers of the human finger nail (Fig. 5). For that reason, the concentration of glycated proteins in the deep layer of the human finger nail is probably a better marker for diabetes mellitus and its complications in comparison with the superficial layer.

In a previous study of our group, ROC analyses of glycated nail proteins yielded an AUC of 0.848 [95% confidence interval (CI): 0.795–0.891] and a cut-off point of 3.14 μmol/g nail (corresponding with a specificity of 93.1% and a sensitivity of 68.9%) for the diagnosis of diabetes mellitus. In older subjects, (age > 60 years), the AUC further increased to 0.885 (95% CI: 0.785–0.949) (specificity: 91.7%, sensitivity: 78.9%; cut-off value = 3.48 μmol/g nail). Patients with diabetic nephropathy and retinopathy showed an increased glycated nail protein concentration [1]. The marked correlation observed between glycated nail proteins and glycated eye lens proteins in the present work confirms the role of glycated nail proteins in the prediction of cataract disease. This relationship was even stronger in diabetes mellitus patients than in nondiabetics, suggesting the impact of AGEs on both eye lens and nail proteins. The presence of lysine residue as a potential glycating site in both eye lens crystallins (α crystallins A and B chains contain 4.1% and 5.7% lysine, β crystallin contains 6.4% lysine and γ crystallin contains 1.2% lysine) [45] and nail keratins could partly explain the correlation observed between the two human tissues. The variation in glycated nail proteins could be explained by its linearly relationship with glycated eye lens proteins and the HbA1c level.

In addition to the presented techniques in this manuscript, an alternative approach with complex tandem mass spectrometry (MS/MS) assays [46–52] could yield a more specific qualitative determination of the glycated and non-glycated nail fractions. The NBT method in this study for measuring fructosamine in nail fragments is based upon the reducing ability of fructosamines in an alkaline solution that can be differentiated from those of other reducing substances (glucose and N-glucosylamine derivatives of labile Schiff bases) [53]. This method has some limitations as only information on the overall level of glycation is obtainable and as there is a lack of glycation site information. A more efficient identification and quantification of non-enzymatically glycated peptides and proteins is feasible by proteomics [46]. However, it was not the authors' intention to develop a high tech analytical method for measuring AGEs, but to develop a practical glycation assay for diabetes detection and monitoring in low-income countries, where glucose determination is hampered by preanalytical conditions (sample transport). The interpretation of HbA1c test results is also hampered by an important local comorbidity (e.g. hemolytic disease, hemoglobinopathies) and the relatively high cost of the test. Epidemiological studies have shown that by 2030, the lion share of diabetics will live in low-income countries. These countries are ill-prepared to tackle the predicted rise of non communicable diseases (e.g. diabetes mellitus). We are convinced that the presented simple methodology may significantly improve diabetes care in low-income countries.

In conclusion, we have demonstrated in the present study that glycation of nail proteins takes place in the deep layer of finger nails, which is in close contact with the blood vessels and the interstitial fluid. Glycation of nail proteins can be regarded as a representative marker for diabetic glycation-associated target organ damage.

## Supporting Information

S1 Dataset Dataset file. (XLSX)
